# Synapsin 1 promotes Aβ generation via BACE1 modulation

**DOI:** 10.1371/journal.pone.0226368

**Published:** 2019-12-12

**Authors:** Masato Maesako, Katarzyna M. Zoltowska, Oksana Berezovska

**Affiliations:** MassGeneral Institute for Neurodegenerative Disease, Alzheimer’s Disease Research Unit, Massachusetts General Hospital, Harvard Medical School, Charlestown, MA, United States of America; Nathan S Kline Institute, UNITED STATES

## Abstract

It has been revealed that β-amyloid (Aβ) is generated and released from the presynaptic terminals in activity-dependent manner. However, molecules modulating the presynaptic Aβ generation remain elusive. Here we test the hypothesis that Synapsin 1 (Syn1) may acts as a modulator of the Aβ production. Using biochemical and Förster resonance energy transfer (FRET)-based imaging approaches we have found that Syn1 knock down decreases, whereas (over)expression of Syn1 in cells increases the Aβ levels. Mechanistically, Syn1 does not seem to affect the activity of Presenilin 1 (PS1)/γ-secretase, PS1 conformation, or the proximity between PS1 and amyloid precursor protein (APP). However, we found that Syn1 is involved in up-regulation of the β-site APP cleaving enzyme 1 (BACE1)/β-secretase activity and increases the APP/BACE1 interaction. Therefore, we conclude that Syn1 may promote Aβ production via the modulation of BACE1.

## Introduction

One of the characteristic pathological features of Alzheimer’s disease (AD) is amyloid plaques deposition in the brain composed of β-amyloid (Aβ). The soluble oligomeric Aβ is believed to be the neurotoxic species disturbing normal synapse function that leads to the memory impairment in AD [1–3]. Aβ derives from the proteolytic processing of the amyloid precursor protein (APP). The β-site APP cleaving enzyme 1 (BACE1), known as β-secretase, cleaves APP at the extramembrane domain, producing the soluble form of APP β (sAPPβ) and membrane-bound APP C-terminus fragment β (APP-CTFβ) [4]. Preseniln 1 (PS1)/γ-secretase subsequently cleaves the APP-CTFβ within the membrane, generating Aβ and APP intracellular domain [5, 6]. Increasing number of evidence suggests that Aβ is released from the presynaptic terminal in a neuronal activity dependent manner [7–10]. Thus, better understanding of the synaptic Aβ regulators would be crucial for potential synapse targeting AD therapeutics.

Several synaptic proteins have been identified as PS1/γ-secretase binding partners and have been shown to affect Aβ production and/or release [11–13]. We have performed mass spectrometry (MS) proteomics screen to identify PS1 binding proteins in mouse brain and have recently shown that a synaptic vesicle-associated protein Synaptotagmin 1 (Syt1), affects PS1/γ-secretase activity and APP processing [13] and regulates Aβ release via direct interaction with PS1 [14]. Synapsin 1 (Syn1) has also appeared in the MS proteomic screen as a novel PS1 interacting protein. Syn1 is known to tether synaptic vesicles to the actin filaments and keep them in the synaptic vesicles reserve pool, thereby modulating neurotransmitter release [15, 16]. However, whether Syn1 may affect the level of Aβ is unknown. In the present study, we used biochemical and Förster resonance energy transfer (FRET)-based imaging techniques to examine the role of Syn1 in the production of Aβ. Here we demonstrate that Syn1 knock down decreases, whereas (over)expression of Syn1 increases the Aβ levels in the conditioned medium. Syn1 modulates neither the activity of PS1/γ-secretase, nor APP and PS1 proximity. Moreover, Syn1 (over)expression does not change PS1 conformation or the Aβ42/40 ratio. On the other hand, we found that Syn1 expression correlates with the strengthened BACE1/β-secretase activity and increases BACE1 proximity interaction with APP. Therefore, we conclude that Syn1 may promote Aβ production via modulation of BACE1.

## Material and methods

### Ethics statements

The protocol for harvesting of mouse brains and neuronal preparation procedure is in compliance with the NIH guidelines for the use of animals in experiments and was approved by the Massachusetts General Hospital Animal Care and Use Committee (2003N000243 and 2006N000026).

### Antibodies and reagents

An anti-FLAG (Wako, Osaka, Japan), anti-sAPPβ (Immuno-Biological Laboratories Co., Ltd, Tokyo, Japan), anti-APP (SIGMA, St. Louis, MO, USA), anti-PS1 loop (Abcam, Cambridge, MA, USA), anti-PS1 NTF (Abcam, Cambridge, MA, USA), anti-BACE1 (EMD Millipore, Burlignton, MA, USA), anti-Syn1 (ECM Biosciences, Versailles, KY, USA) and anti-GAPDH (Cell Signaling Technology, Danvers, CO, USA) antibodies were used in this study. β-Secretase inhibitor was purchased from Millipore-Sigma (Burlignton, MA, USA).

### Cell culture, transfection and lentivirus transduction

Primary neuronal cultures were obtained from cerebral cortex and hippocampus of mouse embryos at gestation day 14–16 (Charles River Laboratories, Wilmington, MA, USA). The neurons were dissociated using Papain Dissociation System (Worthington Biochemical Corporation, Lakewood, NJ, USA) and were maintained for 13–15 days in vitro (DIV) in Neurobasal medium containing 2% B27 supplement, 1% GlutaMax, and 1% Pen/Strep mix (Thermo Fisher Scientific, Waltham, MA, USA). 7W or PS70 CHO cells stably overexpressing human APP or both APP and PS1, respectively, were kind gift from Dr. Dennis Selkoe (Brigham and Women’s Hospital/Harvard Medical School, MA, USA) [17]. For transient transfection into these cells, Lipofectamine 3000 (Thermo Fisher Scientific, Waltham, MA, USA) was used according to the manufacturer’s protocol. Plasmid DNA encoding rat Syn1 with the C-terminal FLAG tag was a kind gift from Dr. Hung-Teh Kao (Brown University, RI, USA). The GFP-PS1-RFP (G-PS1-R) FRET-reporter probe, in which EGFP was fused to the N-terminus of PS1 and RFP was inserted into the loop region between transmembrane domains 6 and 7, was used to monitor the conformational change of PS1 [18]. For the knockdown of Syn1 in primary neurons, shRNA of mouse Synapsin1 (VB160629-1009vbn, 3.09 x 10^8^ TU/mL) or that of scramble control (VB150618-10012, 4.71 x 10^8^ TU/mL) was used (VectorBuilder, Santa Clara, CA, USA). The primary neurons were transduced with the lentiviral particles at multiplicity of infection (MOI) 6 at 4–5 days in vitro (DIV). 12 hours post-infection, the medium was replaced and the neurons were cultured for 5 consecutive days before the analysis.

### Co-immunoprecipitation (co-IP) and Western blotting

Co-IP was performed as described previously [13]. Briefly, 4–6 months old male C57BL/6 mice were euthanized using CO_2_. Whole brains or primary neurons were lysed in a buffer containing 1% Triton X100 (Tx100). The protein concentration was normalized by BCA assay (Thermo Fisher Scientific, Waltham, MA, USA). Conjugation of antibody with Protein G Dynabeads (Thermo Fisher Scientific, Waltham, MA, USA) was performed according to the manufacturer’s protocol. The sample and the Dynabeads-antibody complex were incubated overnight and the immnoprecipitated proteins were eluted in the Elution buffer (50 mM Glycine pH2.8, LDS sample buffer, Reducing Agent; Thermo Fisher Scientific, Waltham, MA, USA).

Western blotting was performed as described previously [19]. Briefly, cells were lysed in a buffer containing 1% Tx100 or 1% Tx100 plus 0.25% NP40. After normalizing protein concentrations by BCA assay, the samples were run on Bis-Tris Gels (Thermo Fisher Scientific, Waltham, MA, USA), the proteins were transferred into nitrocellulose membrane (Thermo Fisher Scientific, Waltham, MA, USA) after the electrophoresis, and detected using corresponding primary and HRP-conjugated secondary antibodies. The blots were developed using Western Lightning Plus ECL reagents (PerkinElmer, Boston, MA, USA).

### Measurement of extracellular sAPPβ and Aβ

The ELISA kits were used to measure the levels of sAPPβ (Immuno-Biological Laboratories Co., Ltd, Tokyo, Japan), Aβ40 and Aβ42 (Wako, Osaka, Japan). Briefly, after the plasmid transfection into PS70 cells or the lenti-virus transduction into primary neurons (see Results), the cells were washed and incubated with fresh Opti-MEM or Neurobasal medium without serum for 6 hours. Then the culture medium was collected and centrifuged at 600 g for 5 minutes. The supernatant was used for the ELISA measurements. Each value recorded in ELISA was normalized to the total protein concentration of the corresponding cell lysate as determined by the BCA protein assay.

### Measurement of BACE1/β-secretase activity

*In vitro* BACE1 activity was measured by using β-secretase activity kit (Abcam, Cambridge, MA, USA) according to the manufacturer’s protocol. Briefly, the kit uses a β-secretase-specific peptide conjugated to two reporter molecules; EDANS and DABCYL. The fluorescent signal from EDANS is quenched by the physical proximity of the DABCYL. Cleavage of the peptide by β-secretase physically separates the two molecules, allowing for the release of a fluorescent signal. Therefore, the level of enzymatic activity in samples is proportional to the level of fluorescence intensity. 24 hours after transfection of Syn1, the PS70 cells were washed by PBS, collected, and lysed using the Extraction Buffer provided in the kit.

### Measurement of PS1/γ-secretase activity

The *in vitro* PS1/γ-secretase activity assay was performed as described previously [20]. Briefly, the crude homogenates of PS70 cells transiently transfected with Syn1 plasmid or control empty vector were homogenized in 20 mM HEPES (pH 7.4) containing protease inhibitor cocktail (Roche, Indianapolis, IN, USA). The lysates were centrifuged at 3000 x g for 15 minutes. The supernatant was centrifuged further at 100,000 x g for 1 hour in a L8-80 M ultracentrifuge equipped with a Ti70.1 rotor (Beckman Coulter. Inc, CA, USA). The pellet was lysed with 1% CHAPSO in the Solubilized Buffer (50mM HEPES, 150mM NaCl, 5mM MgCl2, 5mM CaCl2, pH7.0), followed by centrifugation at 100,000 x g for 1 hour to obtain cell membrane enriched sample. Alpha-Gal C99-FLAG construct was a kind gift from Dr. Satoru Funamoto (Doshisha University, Kyoto, Japan) and recombinant C99-FLAG was generated in Sf9 cells (Thermo Fisher Scientific, Waltham, MA, USA) as described previously [20]. Equal amounts of 1% CHAPSO-solubilized cell membranes were incubated with the recombinant C99-FLAG for 4 hours at 37°C, and the levels of generated Aβ 40 and Aβ 42 were measured by ELISA (Wako, Osaka, Japan).

### Immunocytochemistry (ICC) and Fluorescence Lifetime Imaging Microscopy (FLIM)

ICC and FLIM analysis were performed as described previously [19]. Briefly, after fixation and permeabilization, the cells were immunstained with primary antibodies against APP and PS1 or BACE1. The FLIM assay was used to monitor relative proximity between the fluorescently labeled proteins (e.g. APP and PS1 or BACE) or different domains of the PS1 molecule (PS1 NT and loop). Pulsing Chameleon Ti:Sapphire laser (Coherent Inc., Santa Clara, CA) was used for two-photon fluorophore excitation at 800 nm. The baseline lifetime (*t*1) of the donor fluorophore was measured in the absence of the acceptor fluorophore (negative control, FRET absent). The donor fluorophore lifetimes were recorded using a high-speed photomultiplier tube (MCP R3809; Hamamatsu, Bridgewater, NJ) and a fast time-correlated single-photon counting acquisition board (SPC-830; Becker &Hickl, Berlin, Germany). In the presence of the acceptor fluorophore, excitation of the donor fluorophore results in reduced donor emission energy if the donor and the acceptor are less than 5–10 nm apart (FRET present). This leads to a characteristic shortening of the donor fluorophore lifetime (*t*2). The acquired FLIM data were analyzed using SPC Image software (Becker &Hickl). The percent FRET efficiency (%E_FRET_) was calculated using the following equation: %E_FRET_ = 100*(*t*1—*t*2)/*t*1.

### Statistical analysis

The data comparisons were performed using unpaired Student’s t-test. All values are given as means ± SD. p< 0.05 was considered to indicate a significant difference. All experiments in the study were independently repeated at least three times.

## Results

### Syn1 affects neither PS1/γ-secretase activity nor APP/PS1 proximity

**Fig 1:**
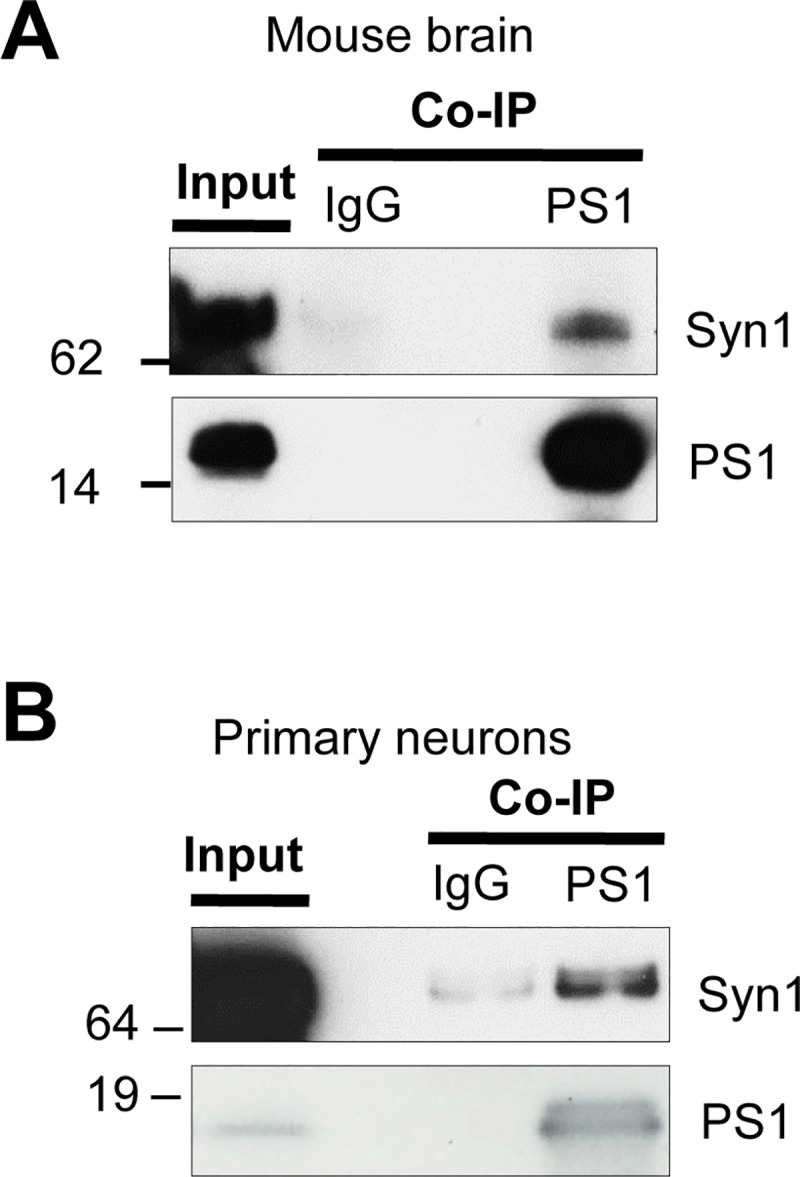
Validation of Syn1/PS1 complex formation by co-immunoprecipitation. Mouse whole brain homogenate (**A**) or primary neuronal lysate (13–15 DIV) (**B**) was immunoprecipitated with an anti-PS1 CT antibody, followed by detection with an anti-Syn1 antibody. Normal rabbit IgG was used as a negative control. Replicated in three independent experiments.

**Fig 2:**
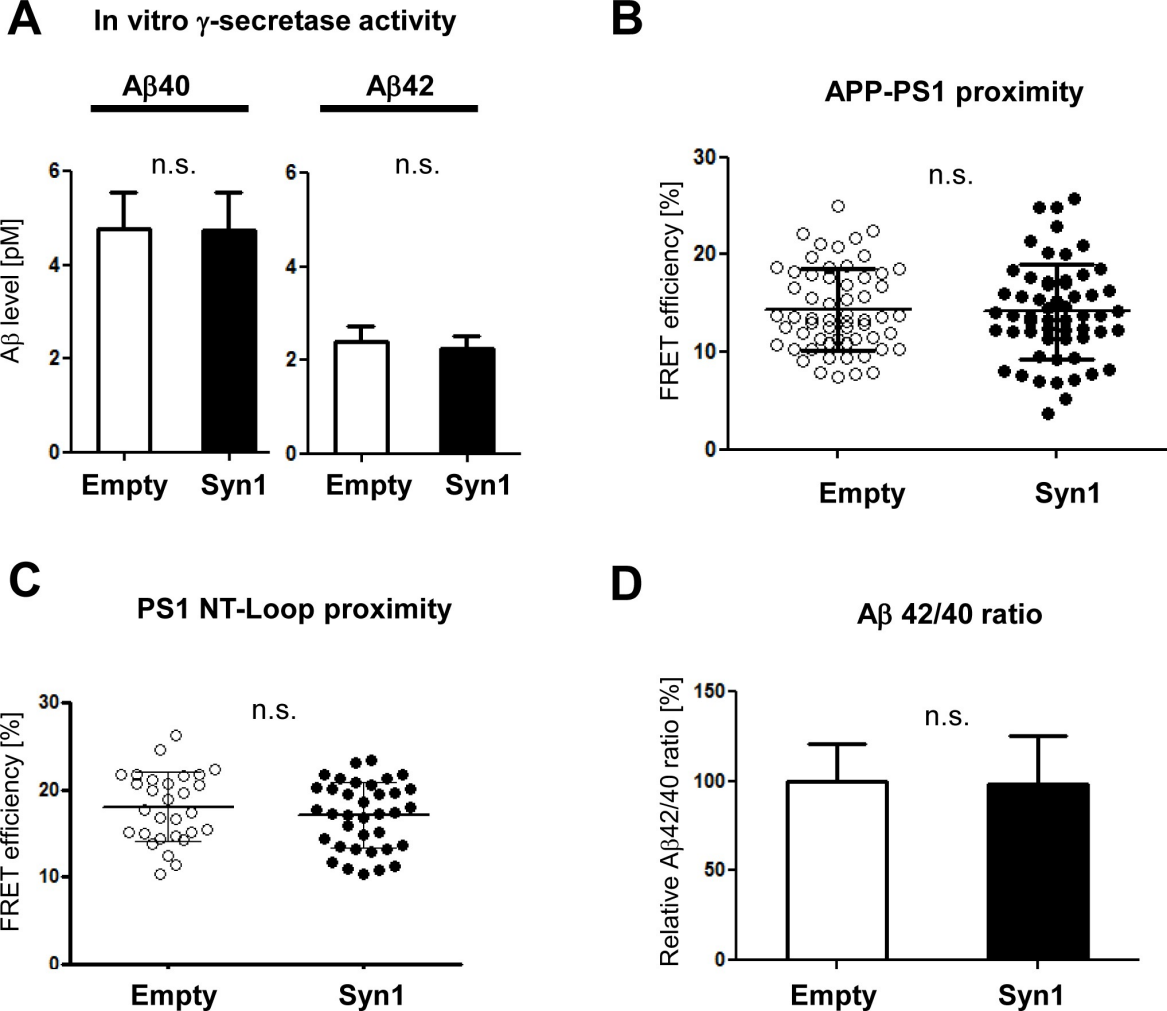
Syn1 does not modulate PS1/γ-secretase. **A.** PS1/γ-secretase activity was determined by a cell-free *in vitro* PS1/γ-secretase assay. The membrane preparations from Syn1 or control vector transfected PS70 cells were incubated with recombinant C99-FLAG, and generated Aβ was detected by ELISA. The production of Aβ40 and Aβ42 in vitro was not affected by Syn1 expression. n = 6 biological replicates from three independent experiments, Student’s t-test, mean ± S.D., n.s. means not significant. **B.** The proximity between APP and PS1 was measured by FLIM in the 7W cells co-transfected with PS1 and either empty vector or Syn1. There was no difference in the APP/PS1 proximity between the Syn1 and the empty vector expressing cells. n = 57–60 cells total from three independent experiments, Student’s t-test, mean ± S.D., n.s. means not significant. **C.** GFP-PS1-RFP was co-transfected with Syn1-FLAG [Syn1(+)] or empty vector [Syn1(-)] into 7W cells, and the proximity between the N-terminus of PS1 (GFP) and the loop (RFP) was measured by FLIM. There was no difference in the PS1 N-term/loop proximity between Syn1 (+) and Syn1 (-) conditions. n = 29–36 cells total from three independent experiments, Student’s t-test, mean ± S.D., n.s. means not significant. **D.** The Aβ42/40 ratio was calculated based on the ELISA measurements of the amount of Aβ40 and Aβ42 secreted by PS70 cells transfected with either Syn1 or empty vector (shown in Fig 4A, 4B and 4C, respectively). n = 15 biological replicates from three independent experiments, Student’s t-test, mean ± S.D., n.s. means not significant.

Our recent MS proteomics screen of mouse brain for PS1 binding proteins [13] identified Syn1 as a novel PS1 binding partner. To verify the PS1-Syn1 interaction at endogenous level, we performed the protein co-immunoprecipitation (co-IP) analysis of the mouse brain and primary neuron lysates. Consistent with the MS proteomics, co-IP revealed PS1-Syn1 complex formation in both mouse brain **(Fig 1A)** and in primary neurons **(Fig 1B)**. To elucidate the potential effect of Syn1 on PS1 function, we first tested the effect of Syn1 expression on the activity of PS1/γ-secretase using an *in vitro* γ-secretase activity assay [20]. Membrane-enriched fractions prepared from PS70 cells expressing Syn1 or empty-vector control were incubated for 4 hrs with the recombinant C99 Flag, an immediate PS1/γ-secretase substrate, at 37°C, and the levels of released Aβ40 and 42 were measured by ELISA. There was no significant difference in the Aβ levels between Syn1 (+) and Syn1 (-) preparations, indicating that the PS1/γ-secretase activity was not changed by the expression of Syn1 in PS70 cells **(Fig 2A)**. Next, we investigated the effect of Syn1 on APP (the substrate) and PS1/γ-secretase (the enzyme) interaction by monitoring APP/PS1 proximity in intact cells using our previously validated FLIM assay [21]. 7W cells co-transfected with Syn1 and PS1 were immunostained with the Alexa488 labelled APP and Cy3-labeled PS1 antibodies. The anti-PS1 antibody was omitted in the negative FRET/FLIM control samples. As expected, significant shortening of the Alexa488 donor fluorophore lifetime in PS1-APP double-immunostained cells, comparing to the FRET negative control, indicates presence of FRET, i.e., close proximity between the fluorescently labelled APP and PS1 molecules. We found that the FRET efficiency was comparable in Syn1 and empty vector expressing cells, suggesting that Syn1 does not significantly affect the proximity between APP and PS1 **(Fig 2B)**. Finally, it is known that the Aβ42/40 ratio closely correlates with the PS1 conformation [18, 22]; thus, we performed FLIM analysis of the PS1 conformation using the PS1 conformation sensitive GFP-PS1-RFP FRET reporter [18]. We found that Syn1 did not change the GFP-PS1 N-terminus to RFP-loop proximity, as a measure of PS1 conformation **(Fig 2C)**, which was consistent with the unchanged Aβ42/40 ratio **(Fig 2D)**.

### Syn1 promotes Aβ production

**Fig 3:**
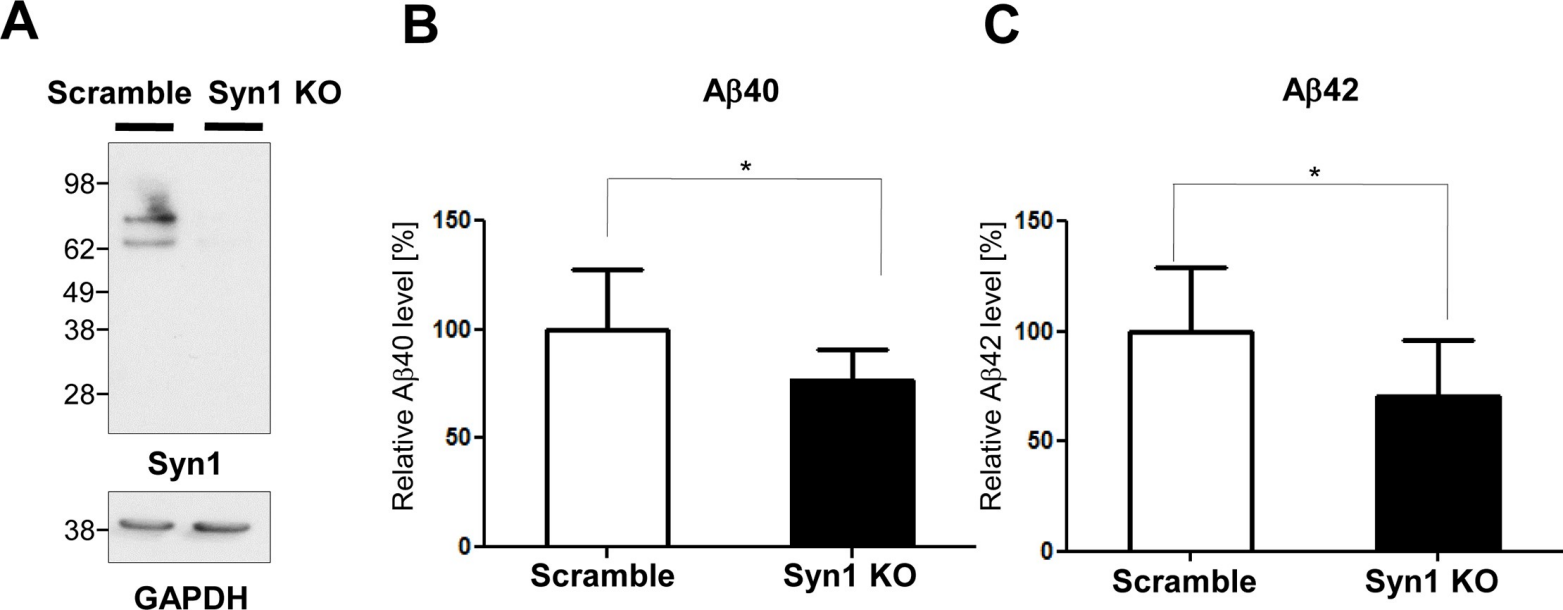
Knockdown of Syn1 decreases Aβ levels in mouse primary neurons. Lentivirus carrying shRNA of Syn1 or control scramble was transduced into mouse primary neurons. Efficient knockdown of Syn1 was verified by Western blotting (two isoforms shown—Syn1a (706 a.a.) and Syn1b (670 a.a.)) (**A**). The levels of Aβ40 (**B**) and Aβ42 (**C**) in the conditioned medium of the Syn1 knockdown primary neurons were measured by ELISA. n = 12 biological replicates from four independent experiments, Student’s t-test, mean ± S.D., *P < 0.05.

**Fig 4:**
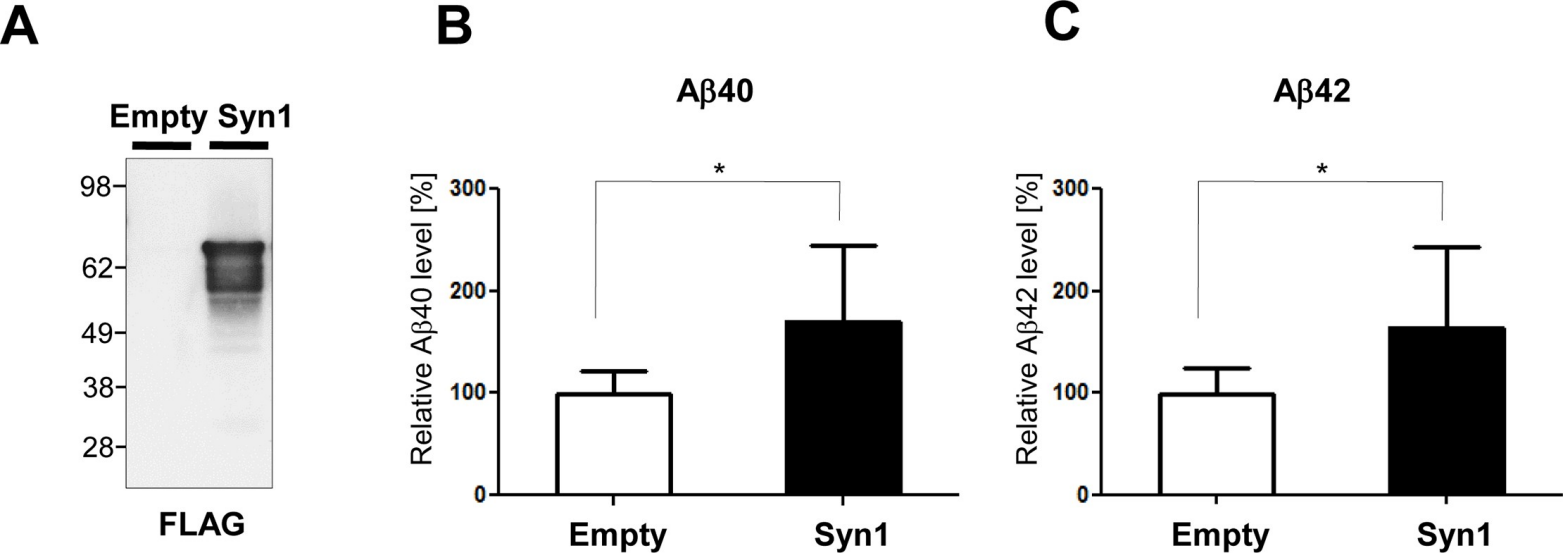
Syn1 expression increases Aβ levels in PS70 cells. Syn1-FLAG or control empty vector was transfected into PS70 cells. Expression of Syn1 was verified by Western blotting with an anti-FLAG antibody (**A**). The levels of Aβ40 (**B**) and Aβ42 (**C**) in the conditioned medium of the Syn1 transfected PS70 cells were measured by ELISA. n = 15 biological replicates from three independent experiments, Student’s t-test, mean ± S.D., *P < 0.05.

To determine if Syn1 may affect Aβ production *per se*, we measured the levels of Aβ40 and Aβ42 in the conditioned medium of primary neurons infected with the lentivirus carrying Syn1 shRNA to suppress the expression of Syn1 **(Fig 3A)**. Despite the lack of Syn1 expression effect on the activity of PS1/γ-secretase or APP and PS1 proximity, we found that the knockdown of Syn1 in primary neurons significantly decreased the levels of both Aβ40 and Aβ42 in the conditioned medium **(Fig 3B and 3C)** without changing the Aβ42/40 ratio (data not shown). Consistent with this finding, the transient expression of Syn1 in PS70 CHO cells significantly increased the levels of Aβ40 and Aβ42 in the conditioned medium **(Fig 4A–4C)**.

### Syn1 enhances BACE1/β-secretase activity

**Fig 5:**
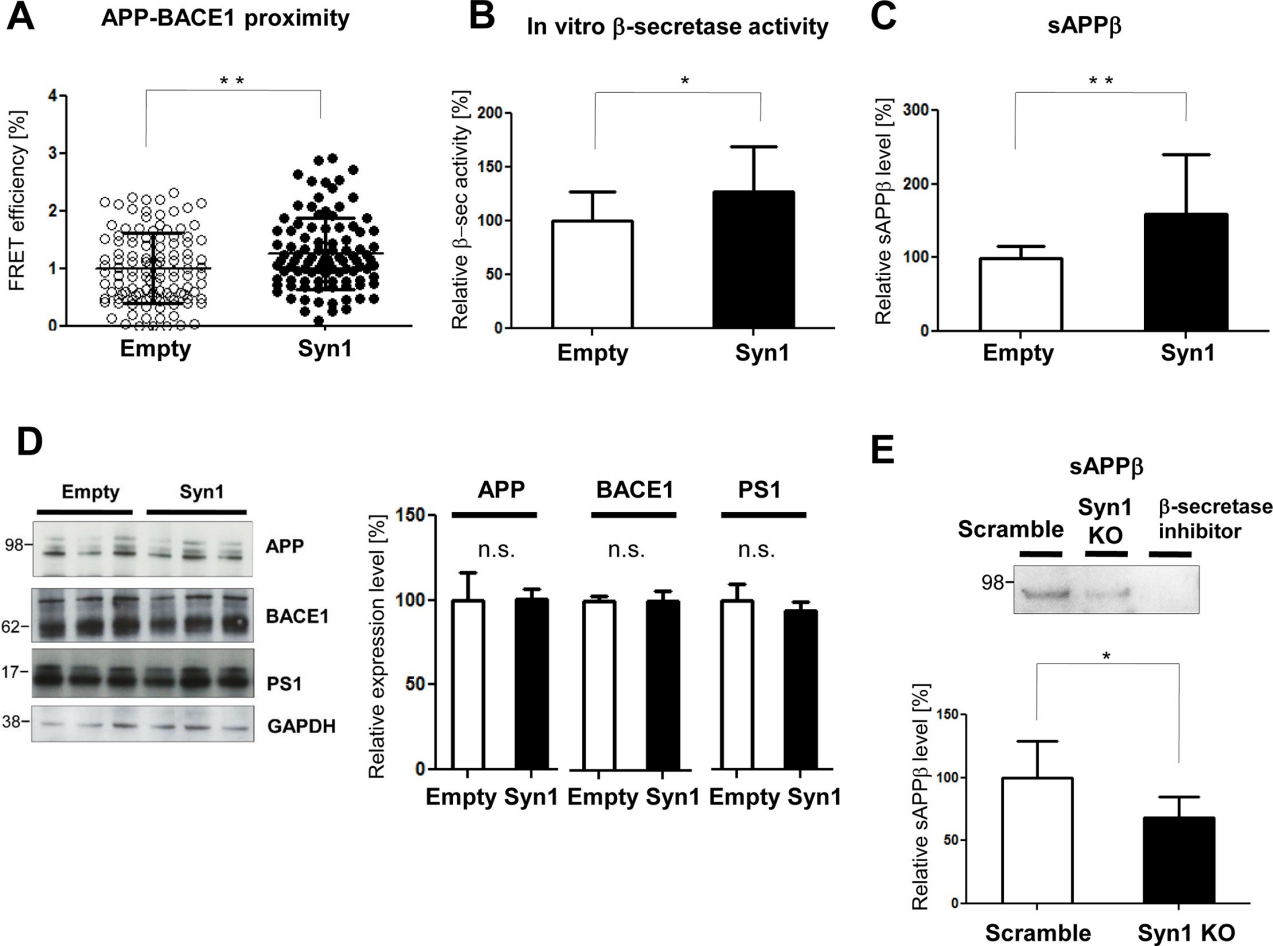
Syn1 modulates BACE1/β-secretase. **A.** The proximity between APP and BACE1 was measured by FLIM in Syn1 and BACE1 co-transfected 7W cells. Closer proximity between APP and BACE1 was observed in Syn1 expressing cells, as indicated by the increased FRET efficiency. n = 99–117 cells total from three independent experiments, Student’s t-test, mean ± S.D., **P < 0.01. **B.** BACE1/β-secretase activity was determined by an *in vitro* BACE1/β-secretase assay in Syn1-FLAG or control vector transfected PS70 cells. Syn1 expression strengthened BACE1/β-secretase activity. n = 15–16 biological replicates from three independent experiments, Student’s t-test, mean ± S.D., *P < 0.05. **C.** The amount of sAPPβ in the conditioned medium of Syn1 expressing PS70 cells was increased compared to that in the empty vector transfected cells, as detected by ELISA. n = 15 biological replicates from three independent experiments, Student’s t-test, mean ± S.D., **P < 0.01. **D.** The expression levels of APP, BACE1, and PS1 in empty vector or Syn1 FLAG transfected PS70 cells were quantified by Western blotting. No change in the expression levels was observed between the Syn1(+) and Syn1(-) conditions. n = 4–8 biological replicates, Student’s t-test, mean ± S.D., n.s. means not significant. **E.** The amount of sAPPβ in the conditioned medium of Syn1 shRNA infected primary neurons was measured by Western blotting. The treatment of cells with a β-secretase inhibitor (1uM) was used as a negative control of the detection. Syn1 knockdown was linked to the decreased sAPPβ level. n = 6 biological replicates from three independent experiments, Student’s t-test, mean ± S.D., *P < 0.05.

To identify the mechanism by which Syn1 expression leads to increased Aβ level, we investigated the effect of Syn1 expression on BACE1. First, we co-transfected Syn1 or control empty vector with BACE1 into 7W CHO cells stably expressing APP, and immnostained the cells with Alexa488-labelled APP and Cy3-labeled BACE1 antibodies. The proximity between APP and BACE1 was measured by the FLIM analysis in intact cells. Syn1 expression resulted in significantly increased APP/BACE1 proximity **(Fig 5A**), suggesting that the APP/BACE1 interaction was enhanced by Syn1 expression. We next performed an *in vitro* BACE1/β-secretase activity assay in PS70 CHO cells and found that Syn1 strengthens the activity of endogenous BACE1 **(Fig 5B)**. Consistent with the results from the FLIM assay and *in vitro* β-secretase activity assay, the level of sAPPβ was significantly increased in the conditioned medium of Syn1 expressing PS70 cells **(Fig 5C)**. Of note, the effect of Syn1 on BACE1/β-secretase activity and interaction with APP was not due to the altered expression levels of APP and BACE1, as Syn1 did not significantly change the level of APP and BACE1 protein in these cells **(Fig 5D)**. On the other hand, Syn1 knockdown in primary neurons decreased the level of sAPPβ, suggesting that BACE1 activity is tightly associated with the Syn1 expression in neurons **(Fig 5E)**.

## Discussion

Local production and accumulation of synaptic Aβ leading to synaptic dysfunction and neurodegeneration has been well documented [3, 7, 23–25]. Though the molecules affecting activity dependent Aβ production in the post-synapse have been reported [12, 25], molecular modulators of the pre-synaptic Aβ are less defined. Here we investigate the role of presynaptic protein: Syn1 on Aβ generation. We show that Syn1 enhances Aβ production by strengthening BACE1/β-secretase activity and promoting the APP-BACE1 interaction.

The level of synaptic Aβ is dynamically influenced by neuronal activity and is believed to be primarily related to the synaptic vesicle exocytosis from the pre-synaptic terminals [7]. Accordingly, the electron microscopy studies have verified presence of the key molecules necessary for the Aβ production—APP [26], BACE1 [27], and, PS1 [13] in the synaptic vesicles. Synaptic vesicles (SV) reside in three different pools: the reserve pool makes up ~80–90% of the total pool, the recycling pool is significantly smaller (~10–15%), and the readily releasable pool consists of a few vesicles (~1%) that seem to be docked to the pre-synaptic membrane and primed for the release [28]. However, exactly where/in what SV pool Aβ is generated and how it is regulated remains unclear. Syn1 is known to tether synaptic vesicles to the actin filaments, thus preventing them from migrating and keeping them in the SV reserve pool [15, 16]. Neuronal activity and consequent Ca^2+^ influx triggers phosphorylation of the Syn1 [29]. This phosphorylation induces Syn1/actin filaments disintegration [15, 16], leading to release of the synaptic vesicles from the reserve pool. Concurrently, Ca^2+^ binding to another synaptic vesicle associated protein, Synaptotagmin 1 (Syt1), triggers the migration of synaptic vesicles towards the plasma membrane, with Syt1 mediating the docking, fusion and exocytosis of the readily releasable synaptic vesicles at the synaptic cleft [30–32].

We have uncovered that both Syn1 and Syt1 interact with the PS1/γ-secretase, and recently reported that Syt1 interaction with PS1 is triggered by Ca^2+^ influx [13, 14]. It has also been shown that APP interacts with a number of pre-synaptic proteins and has been proposed that APP is processed by BACE1 within the synaptic vesicles [33, 34]. Given that the majority of synaptic vesicles reside in the Syn1-tethered reserve pool, and as the present study found that Syn1 modulates BACE1/β-secretase activity and fosters APP-BACE1 interaction, we suggest that APP might be cleaved by BACE1/β-secretase in the reserve pool, thus further refining the presynaptic location of APP cleavage by BACE1. On the other hand, Ca^2+^ triggered Syt1 interaction with PS1/γ-secretase has been reported to positively modulate γ-secretase activity, Aβ generation and release at the pre-synapse [14]. Taken together, these finding suggest that Syn1 modulates BACE1/β-secretase mediated APP-CTFβ generation in the synaptic vesicles of the reserve pool. Then the APP-CTFβ is further processed by PS1/γ-secretase to produce and release Aβ during the activity-driven exocytosis, and Syt1 may be involved at this stage.

Surprisingly, while PS1/γ-secretase was not significantly affected by Syn1 expression, Syn1 promoted the activity of BACE1/β-secretase. This seeming discrepancy could be explained, at least partially, by recently described physiological interaction between the β- and γ-secretases existing as a distinct β-secretase/γ-secretase enzymatic complex in the brain [35, 36]. It is plausible that Syn1 binding to PS1 may affect BACE1 functioning within the complex. Our study also shows that Syn1 does not seem to affect BACE1 expression but strengthens the BACE1/β-secretase activity. However, the mechanism behind this is unclear. Structural studies established that the active site of BACE1 is covered by a flexible antiparallel β-hairpin, which is believed to regulate substrate access to the active site [37–39]. Furthermore, BACE1 activity is tightly associated with the pH [40] and, importantly, the flexible antiparallel β-hairpin of active BACE1 displays open conformation, which enables the access of substrates into the catalytic site in a pH dependent manner [39]. It is possible that Syn1 may “force” the positioning of BACE1 in its preferable pH environment, thus inducing the BACE1 substrate accessible conformational change. Consistent with this idea, we found that Syn1 (over)expression is linked to decreased proximity between the BACE1 and APP. A previous study has reported that decreased proximity between APP and BACE1 is linked to increased production of Aβ [41]. In the present study, we knocked down Syn1 expression using shRNA to examine the role of Syn1 on Aβ production in primary neurons. Since it has been reported that Syn1 knockdown may impair neuronal functions, such as neurite outgrowth, synaptogenesis, and/or synaptic transmission [42], we could not rule out the possibility that these altered functions could have subsequently affected APP/BACE1 localization and processing, and thus Aβ production.

## Conclusions

In summary, the current study reports Syn1 as a novel synaptic regulator of Aβ, and demonstrates that Syn1 promotes Aβ production by strengthening BACE1/β-secretase activity and inducing APP-BACE1 interaction. In addition, our finding further refine the specific presynaptic location/synaptic vesicle pools where APP is processed and Aβ is generated, and point to SV associated proteins, Syn1 and Syt1, as modulators of the pre-synaptic Aβ generation and release.

## Supporting information

S1 Fig The original uncropped and unadjusted Western blotting images and all individual data points within column graphs. (PDF)

## Abbreviations

Aβ: β-amyloid
AD: Alzheimer’s disease
APP: Amyloid precursor protein
BACE1: β-site APP cleaving enzyme 1
co-IP: co-immunoprecipitation
FRET: Förster resonance energy transfer
PS1: Presenilin 1
sAPPβ: soluble form of APP β
Syn1: Synapsin 1
Syt1: Synaptotagmin 1
