# Supplementary material for: Synapsin 1 promotes Aβ generation via BACE1 modulation
Source: PLoS One. 2019 Dec 12;14(12):e0226368. doi: 10.1371/journal.pone.0226368 (PMC6907790; doi:10.1371/journal.pone.0226368)
Supplement: S1 Fig — (PDF) [file pone.0226368.s001.pdf]

Figure 1A

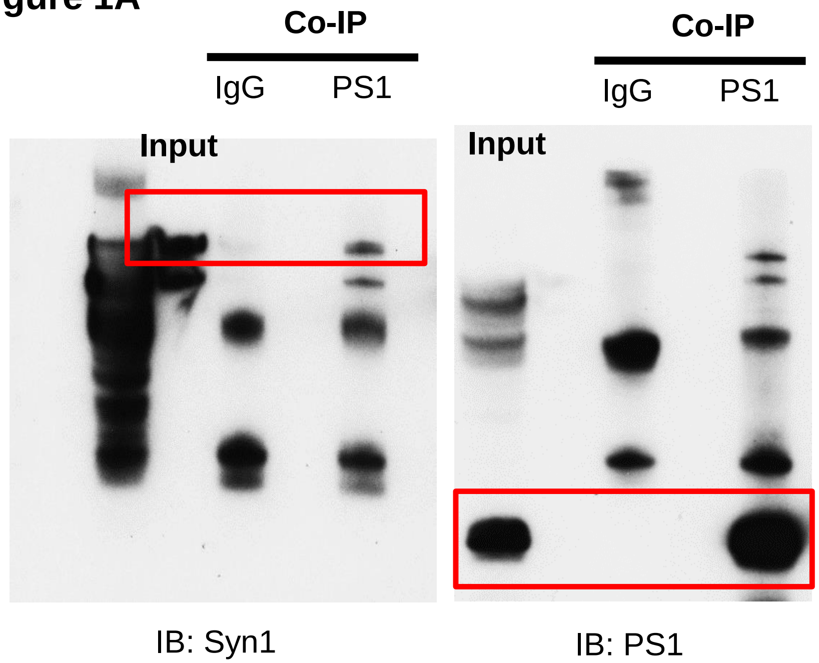

Figure 1B

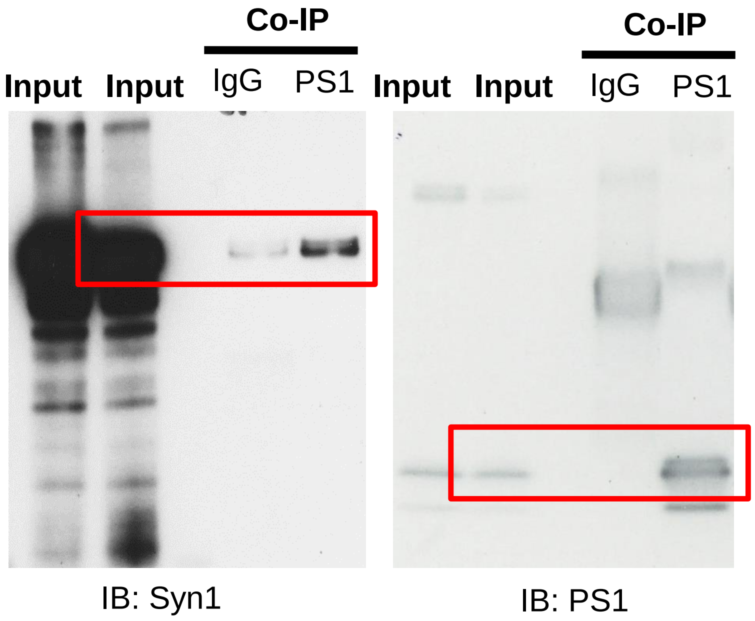

Figure 3A

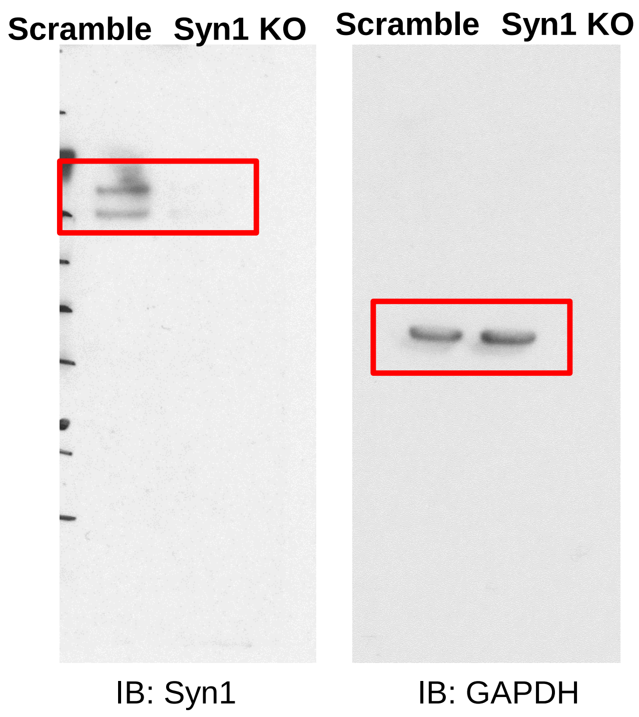

Figure 4A

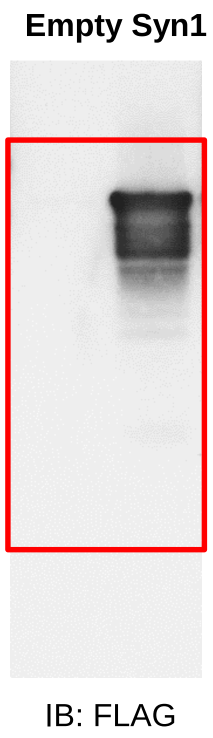

Figure 5D

Empty **Syn1**

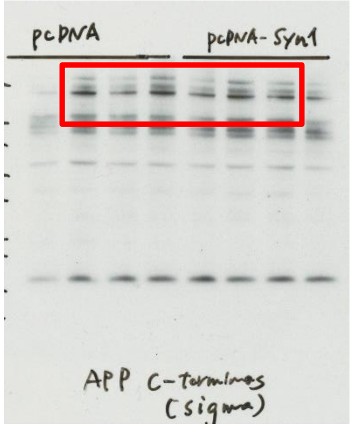

IB: APP

Empty **Syn1**

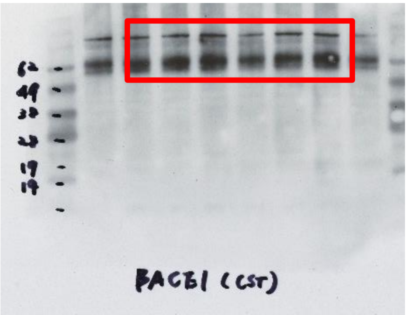

IB: BACE1

Empty **Syn1**

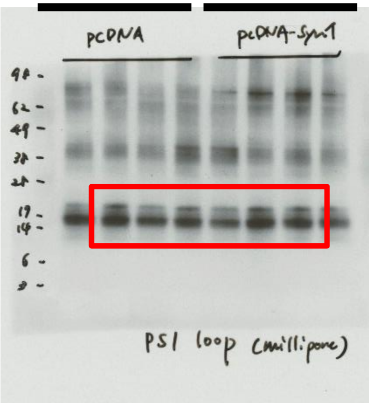

IB: PS1

Empty **Syn1**

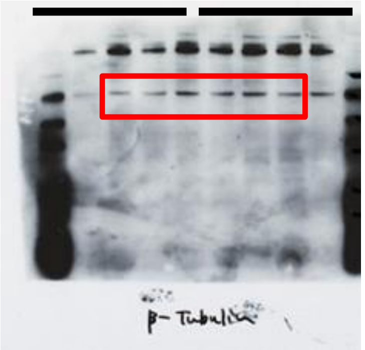

IB: beta-tubulin

Figure 5E

Scramble **Syn1 KO** **β-secretase inhibitor**

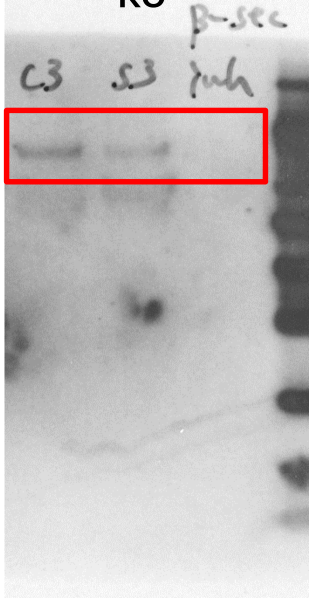

IB: sAPPβ

Figure 2A

Aβ40

Empty	Syn1
4.446252	5.475477
3.664041	3.622872
4.693266	3.952224
4.775604	4.775604
6.093012	5.516646
4.981449	5.187294

Aβ42

Empty	Syn1
1.92234	1.60195
1.83876	1.72732
3.71931	2.64670
1.99199	2.39596
2.96709	1.82483
2.06164	3.30141

Figure 2D

Aβ42/40 ratio

Empty	Syn1
77.508	82.727
130.121	85.915
93.079	78.315
119.950	130.918
79.341	174.249
101.969	94.429
100.680	92.359
114.613	72.572
77.119	99.300
100.154	88.122
105.464	119.257
75.510	87.636
83.182	63.853
144.277	101.204
97.032	104.951

Figure 3B

Aβ40

Scramble	Syn1 KO
71.60133	74.75469
140.21250	73.11405
88.18618	86.01777
66.45908	90.29547
73.13449	87.25593
160.40640	38.70266
108.47890	84.64423
84.39613	69.56506
107.12500	76.16484
104.83870	93.15940
94.91822	74.38856
100.24300	74.71276

Figure 3C

Aβ42

Scramble	Syn1 KO
88.58217	81.56780
88.79622	70.00000
122.62160	84.95385
44.54634	22.04910
87.50804	59.16565
167.94560	24.48500
110.22540	76.11932
77.72334	73.16310
112.05130	69.29125
109.49680	112.79900
90.43928	90.38748
100.06390	83.33131

Figure 4B

Aβ40

Empty	Syn1
78.455	113.173
84.517	103.390
117.671	131.005
103.734	136.188
115.622	92.377
107.105	155.994
85.600	121.105
54.933	212.719
149.698	380.476
94.973	243.416
107.690	184.156
112.164	131.296
74.749	181.102
104.911	144.038
108.175	226.160

Figure 4C

Aβ42

Empty	Syn1
61.241	94.289
110.754	89.459
110.304	103.325
125.313	179.560
92.387	128.618
112.496	151.730
88.772	115.212
64.852	159.014
118.914	389.166
97.978	220.950
116.987	226.219
84.023	114.149
61.684	114.720
150.161	144.616
104.132	235.473

Figure 5B

in vitro β-secretase activity

Empty	Syn1
107.20000	142.40000
67.20000	120.00000
125.60000	94.40000
99.20000	236.00000
102.40000	68.80000
88.05310	154.86730
111.06190	147.34510
107.52210	154.86730
92.92035	132.30090
99.55752	81.85841
160.12660	90.26549
133.54430	163.29110
68.35443	131.64560
86.07595	137.97470
53.16456	91.13924
	81.01266

Figure 5C

sAPPβ

Empty	Syn1
101.476	89.803
94.880	103.149
102.493	101.799
98.571	109.942
102.579	93.663
91.575	165.522
93.071	123.914
62.689	258.528
133.460	386.313
104.937	234.244
114.268	178.770
104.592	112.765
85.663	138.143
103.384	113.809
106.361	181.160

Figure 5D

APP		BACE1		PS1	
Empty	Syn1	Empty	Syn1	Empty	Syn1
68.796080	102.734600	96.651860	97.960930	118.472900	101.083700
112.034200	112.542200	100.249600	101.425700	104.062300	86.429860
93.947430	104.215200	99.821330	107.799000	93.561150	98.712010
125.222300	98.704120	103.277300	93.225690	83.903630	88.724010
103.764500	94.360450			97.324650	95.879460
97.162150	97.887570			102.249800	96.515380
102.533400	99.692920			100.398700	94.888400
96.539940	92.137720			100.026900	85.992520

Figure 5E

Scramble	Syn1 KO
74.13012	65.49796
90.16215	78.45433
135.70770	94.69215
85.18041	62.71088
76.79793	43.63808
138.02170	63.01820
